# Characterization of Maltase and Sucrase Inhibitory Constituents from *Rhodiola crenulata*

**DOI:** 10.3390/foods8110540

**Published:** 2019-11-02

**Authors:** Wen-Tai Li, Yu-Hsuan Chuang, Jung-Feng Hsieh

**Affiliations:** 1 National Research Institute of Chinese Medicine, Ministry of Health and Welfare, Taipei 11221, Taiwan; lwt0220@nricm.edu.tw; 2 Department of Food Science, Fu Jen Catholic University, Taipei 242, Taiwan; d88320001@pchome.com.tw

**Keywords:** maltase, sucrase, inhibitor, inhibition kinetics assay, *Rhodiola crenulata*

## Abstract

The inhibitory properties of epicatechin-(4*β*,8)-epicatechingallate (B2-3’*-O-*gallate), epicatechin gallate (ECG), and epicatechin (EC) isolated from *Rhodiola crenulata* toward maltase and sucrase were investigated. The half-maximal inhibitory concentration (IC_50_) values for maltase were as follows: B2-3’-*O*-gallate (1.73 ± 1.37 μM), ECG (3.64 ± 2.99 μM), and EC (6.25 ± 1.84 μM). Inhibition kinetic assays revealed the inhibition constants (*K*i) of the mixed-competitive inhibitors of maltase, as follows: B2-3’-*O*-gallate (1.99 ± 0.02 μM), ECG (3.14 ± 0.04 μM), and EC (7.02 ± 0.26 μM). These compounds also showed a strong inhibitory activity toward sucrase, and the IC_50_ values of B2-3’-*O*-gallate, ECG, and EC were 6.91 ± 3.41, 18.27 ± 3.99, and 18.91 ± 3.66 μM, respectively. Inhibition kinetic assays revealed the inhibition constants (*K*i) of the mixed-competitive inhibitors of sucrase as follows: B2-3’-*O*-gallate (6.05 ± 0.04 μM), ECG (8.58 ± 0.08 μM), and EC (13.72 ± 0.15 μM). Overall, these results suggest that B2-3’-*O*-gallate, ECG, and EC are potent maltase and sucrase inhibitors.

## 1. Introduction

Over the past three decades, the number of people with diabetes mellitus has quadrupled, with the result being that diabetes mellitus is now the ninth leading cause of death worldwide. Approximately 1 in 11 adults have diabetes mellitus, and roughly 90% of those cases are type 2. The most pronounced symptom of diabetes mellitus is an abnormal postprandial increase in blood glucose levels [1,2]. It is widely believed that control over postprandial hyperglycemia is crucial to the effective treatment of diabetes mellitus [3]. It is well known that dietary carbohydrates are broken down into monosaccharides by hydrolytic enzymes, α-glucosidases, which are absorbed into the intestinal brush border membrane. The final step of carbohydrate digestion can be catalyzed by α-glucosidases. Furthermore, a number of physiologically important enzymes are involved in the process of digesting dietary carbohydrates [4,5]. Thus, one approach to controlling this digestion process involves delaying the absorption of glucose by inhibiting α-glucosidase activity [6,7].

α-Glucosidases are divided into four hydrolase types, i.e., maltase (Enzyme Commission (EC) 3.2.0.20), sucrase (EC 3.2.1.48), glucoamylase (EC 3.2.1.3), and isomaltase (EC 3.2.1.10). These four enzymes form two complexes with different substrate specificities, maltase–glucoamylase and sucrase–isomaltase complexes [8]. Among them, maltase is the major enzyme responsible for the digestion and absorption of dietary starch, whereas sucrase can hydrolyze sucrose [9]. Maltase is a membrane-bound enzyme that binds to microvilli on intestinal enterocytes. It can hydrolyze the α-1,4 linkages of maltose residues to release a single glucose molecule [10,11]. In addition, sucrase is localized in the brush border membranes of the small intestine. This enzyme can hydrolyze the α-1,2 linkages of sucrose to release glucose and fructose [12]. By acting against these enzymes in the gut, maltase and sucrase inhibitors reduce postprandial glucose levels by restraining the liberation of glucose from oligosaccharides and disaccharides [4]. Nijpels et al. [13] reported that diabetes could be managed by reducing the impact of glycemia by inhibiting maltase and sucrose activity through the regular consumption of the antihyperglycemic drug acarbose. In one intervention study, the daily consumption of acarbose for a period of three years was shown to reduce the risk of developing type 2 diabetes by 6%, compared to a control group that was not consuming acarbose.

In a study by Kwon et al. [14], water extracts of *Rhodiola crenulata* (*R. crenulata*) were shown to significantly reduce the inhibitory activity of α-glucosidase. The genus *Rhodiola* L. (Crassulaceae) comprises approximately 90 species found throughout the world, and more than 70 species are found in the western and northern regions of Asia. Among these, *R. crenulata*, is an important member of the Crassulaceae family. For centuries, the roots of *R. crenulata* have been used as a health supplement. It is possible that these extracts could be used as a supplement for postprandial hyperglycemia associated with diabetes mellitus. In our previously study, we isolated and characterized α-glucosidase inhibitory constituents, including epicatechin-(4*β*,8)-epicatechingallate (B2-3’-*O*-gallate), epicatechin gallate (ECG), and epicatechin (EC) from *R. crenulata* [15]. These results clearly demonstrated the strong inhibitory effects of B2-3’-*O*-gallate, ECG, and EC against α-glucosidase activity.

The above potent maltase and sucrase inhibitors were considered for use in treating diabetes mellitus. Although the inhibition of α-glucosidases by B2-3’-*O*-gallate, ECG, and EC has previously been reported, the inhibitory properties of B2-3’-*O*-gallate, ECG, and EC toward maltase and sucrase have not been examined. Thus, this paper describes the inhibition kinetics of B2-3’-*O*-gallate, ECG, and EC toward maltase and sucrase. Our primary objective in this study was to elucidate the inhibitory effects of 2-3’-*O*-gallate, ECG, and EC on maltase and sucrase.

## 2. Materials and Methods 

### 2.1. Preparation of B2-3’-O-gallate, ECG, and EC from R. crenulata

Dried roots of *R. crenulata* were obtained from a traditional Chinese medicine pharmacy in Chiayi in south Taiwan. The authenticity was confirmed by Dr. Hsiang-Wen Tseng (Industrial Technology Research Institute, Taiwan) using DNA sequencing technology and an internal transcribed spacer sequence database. Isolates of B2-3’-*O*-gallate, ECG, and EC were prepared from a crude extract of *R. crenulata* in accordance with the methods reported by Chu et al. [15]. Briefly, a total of 90 g *R. crenulata* roots was milled and extracted using distilled water (900 mL). After centrifugation at 12,000 × *g* for 15 min, the supernatant was collected and freeze-dried to yield 16 g of water extract. The extract that displayed IC_50_ value for maltase was 5.23 ± 0.18 μg/mL, whereas the extract that displayed IC_50_ value for sucrase was 21.42 ± 0.45 μg/mL. The water extract underwent column chromatography on a vacuum manifold using solid-phase extraction (SPE) cartridges. Each sample (50 mg) was chromatographed on a SPE cartridge. The samples were then eluted stepwise using 0%, 10%, 20%, 30%, and 40% methanol in distilled water. A total of 5 fractions, one for each methanol elution (20 mL), were collected. The samples were concentrated using a rotary evaporator and then freeze-dried. The total yield of the fractions was 92.4%, whereas the yields of the individual fractions were as follows: 0% elution (67.6%), 10% elution (16.5%), 20% elution (5.1%), 30% (2.3%), and 40% (0.9%). The 30% fraction was redissolved in distilled water and subjected to column chromatography on a HPLC high-performance liquid chromatography (HPLC) system comprising a pump (PU-980, JASCO, Tokyo, Japan) and a detector (UV-970, JASCO) with a C18 packed column (4.6 mm × 250 mm, 5 μm Spherical, Dikma Technologies Inc.). A gradient elution from H_2_O/acetonitrile (86:14) to H_2_O/acetonitrile (72:28) was used to isolate B2-3’-*O*-gallate, ECG, and EC. Elution began with a solvent flow rate of 1 mL/min for the collection of B2-3’-*O*-gallate, ECG, and EC. The total yields were as follows: B2-3’-*O*-gallate (8.7 mg), ECG (6.1 mg), and EC (7.6 mg).

### 2.2. Maltase Activity Assay

Maltase activity was characterized using the methods outlined by Adisakwattana et al. [16] with slight modifications. Maltose and maltase derived from *Saccharomyces cerevisiae* (*S. cerevisiae*) were obtained from Sigma Chemical Co. (St. Louis, MO, USA). A reaction mixture was formulated from 30 μL of maltose (86.3 mM) with 20 μL of maltase (0.33 units/mL) and 50 μL of phosphate buffer (100 mM, pH 7.0). The mixture was incubated at 37 °C for 10 min and then at 100 °C for a further 10 min to stop the reaction. The glucose concentrations released by the reaction mixture were determined via the glucose oxidase method (glucose assay kit, Sigma Chemical Co., St. Louis, MO, USA) using a VersaMax microplate reader (Molecular Devices Corporation, Sunnyvale, CA, USA) at an absorbance wavelength of 540 nm.

### 2.3. Maltase Inhibitory Activity of B2-3’-O-gallate, ECG, EC, and Quercetin

In this study, we focused on B2-3’-*O*-gallate, ECG, EC, and quercetin, with quercetin (Sigma Chemical Co., St. Louis, MO, USA) as positive controls. All of the samples were assessed in terms of maltase inhibitory activity under a range of concentrations (0–30 μM). Maltase inhibitory activity was evaluated by dissolving the samples directly in phosphate buffer solution (100 mM, pH 7.0). Note that each sample was assayed in triplicate. Inhibitory activity was estimated in terms of IC_50_ values based on percent inhibition, as follows:

percent inhibition (%) = [(*A_without sample_* − *A_with sample_*)] × 100%/*A_without sample_* (1)

### 2.4. Sucrase Activity Assay

Estimates of sucrase activity were obtained using the method outlined by Akkarachiyasit et al. [17] with slight modifications. Sucrose and sucrase derived from *Leuconostoc mesenteroides* were obtained from Sigma Chemical Co. (St. Louis, MO, USA). The reaction mixture comprised 40 μL of sucrose (480 mM) with 10 μL of sucrase (0.33 units/mL) in 50 μL of phosphate buffer solution (100 mM, pH 7.0). The mixtures were incubated at 37 °C for 60 min and then at 100 °C for a further 10 min to stop the reaction. The glucose concentrations released from the reaction mixtures were estimated via the glucose oxidase method (glucose assay kit, Sigma Chemical Co., St. Louis, MO, USA) using a VersaMax microplate reader (Molecular Devices Corporation, Sunnyvale, CA, USA) at an absorbance wavelength of 540 nm.

### 2.5. Sucrase Inhibitory Activity of B2-3’-O-gallate, ECG, EC, and Quercetin

In this study, we focused on B2-3’-*O*-gallate, ECG, EC, and quercetin, with quercetin (Sigma Chemical Co., St. Louis, MO, USA) as positive controls. All samples were assessed in terms of sucrase inhibitory activity under a range of concentrations (0–50 μM). Inhibitory activity was assessed by dissolving samples directly in phosphate buffer solution (100 mM, pH 7.0). Inhibitory activity was estimated in terms of IC_50_ values based on percent inhibition, as follows:

percent inhibition (%) = [(*A_without sample_* − *A_with sample_*)] × 100%/*A_without sample_* (2)

### 2.6. Lineweaver–Burk Plots and Dixon Plots

Lineweaver–Burk plot analysis was used to identify the mode of inhibition of B2-3’-*O*-gallate, ECG, EC, and quercetin on maltase and sucrase. Kinetics assays were conducted using substrates of various concentrations with and without inhibitors. Maltose was used as a substrate for maltase, where the initial velocity was expressed as the rate of absorbance at 540 nm per min. Sucrose was used as a substrate for sucrase, where the initial velocity was expressed as the rate of absorbance at 540 nm per 18 min. Dixon plot analysis was used to determine the competitive inhibition constant (*K*i) and uncompetitive inhibition constant (*K*i’). *K*i was used as the equilibrium constant for the inhibition of binding to maltase and sucrase, whereas *K*i’ was used as the equilibrium constant for the inhibition of binding to maltase–maltose and sucrase–sucrose complexes. The *K*i and *K*i’ values of the maltase inhibitors were derived from Dixon plots in accordance with the methods described by Cornish-Bowden [18].

### 2.7. Statistical Analysis

Data are expressed as the mean ± standard deviation. All data analysis was performed using Statistical Analysis System (SAS software release 9.4 for Windows, version 13.2, SAS Institute, Inc., Cary, NC, USA). Statistically significant differences between treatment groups were determined using one-way ANOVA followed by Duncan’s multiple range test. Three replicates of each sample were assayed, and the level of significance was set at *p* < 0.05.

## 3. Results and Discussion

### 3.1. Maltase and Sucrase Inhibitory Activity of B2-3’-O-gallate, ECG, EC, and Quercetin

**Figure 1:**
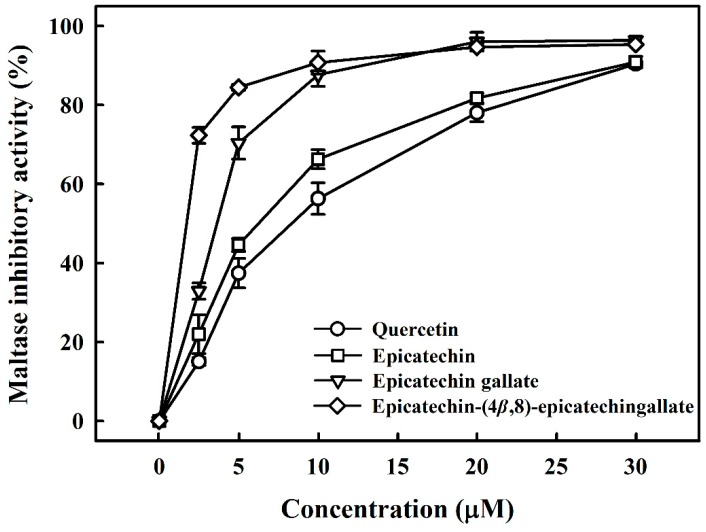
Inhibitory effects of epicatechin-(4*β*,8)-epicatechingallate (B2-3’-*O*-gallate), epicatechin gallate (ECG), epicatechin (EC), and quercetin on maltase. Each value is represented as the mean ± standard deviation of triplicate measurements.

Potent maltase and sucrase inhibitors have been considered for use for treating diabetes mellitus [19]. Thus, we investigated the inhibition of maltase and sucrase by B2-3’-*O*-gallate, ECG, and EC derived from *R. crenulata*. Figure 1 compares the inhibitory activity of B2-3’-*O*-gallate, ECG, and EC with that of quercetin, which is a leading candidate for the treatment of diabetes mellitus [20]. Quercetin was used in this study as a standard inhibitor, which reduces the hydrolysis of maltose by inhibiting the activity of maltase. Wang et al. [21] reported that quercetin showed high inhibitory activity, with an IC_50_ value of 4.8 ± 0.4 mM against rat maltase. We assessed the inhibitory effects of B2-3’-*O*-gallate, ECG, EC, and quercetin on maltase at various concentrations. The addition of B2-3’-*O*-gallate, ECG, EC, and quercetin at a concentration of 10 µM was shown to significantly enhance maltase inhibition, as follows: B2-3’-*O*-gallate (90.7%), ECG (87.7%), EC (66.3%), and quercetin (56.3%). 

**Figure 2:**
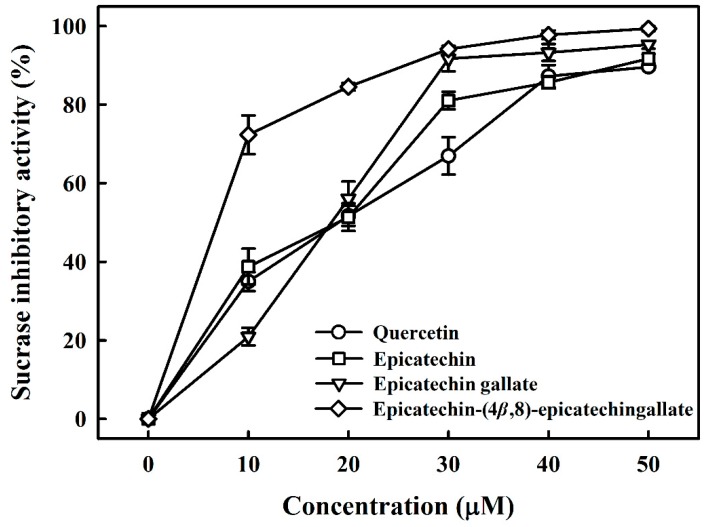
Inhibitory effects of epicatechin-(4*β*,8)-epicatechingallate, epicatechin gallate, epicatechin, and quercetin on sucrase. Each value is represented as the mean ± standard deviation of triplicate measurements.

Table 1 lists the structures of B2-3’-*O*-gallate, ECG, EC, and quercetin. The IC_50_ values for maltase were as follows: B2-3’-*O*-gallate (1.73 ± 1.37 µM), ECG (3.64 ± 2.99 µM), EC (6.25 ± 1.84 µM), and quercetin (8.33 ± 3.91 µM). The strength of the IC_50_ values was in the following order: B2-3’-*O*-gallate < ECG < EC < quercetin. We also compared the inhibitory activities of B2-3’-*O*-gallate, ECG, EC, and quercetin toward sucrase. As shown in Figure 2, the addition of B2-3’-*O*-gallate, ECG, EC, and quercetin at a concentration of 20 µM was shown to significantly enhance sucrase inhibition, as follows: B2-3’-*O*-gallate (84.6%), ECG (56.1%), EC (51.4%), and quercetin (51.7%).

As shown in Table 1, the IC_50_ values for sucrase were as follows: B2-3’-*O*-gallate (6.91 ± 3.41 µM), ECG (18.27 ± 3.99 µM), EC (18.91 ± 3.66 µM), and quercetin (18.98 ± 2.53 µM). The strength of the IC_50_ values was in the following order: B2-3’-*O*-gallate < ECG < EC < quercetin. We can see that the inhibitory effects of quercetin on sucrase were far less pronounced than those of B2-3’-*O*-gallate, ECG, and EC (*p* < 0.05). There has already been considerable research into the use of these polyphenols as maltase and sucrase inhibitors as an alternative to pharmaceutical treatments such as acarbose for the management of type 2 diabetes. Pyner et al. [22] reported that the IC_50_ values of polyphenol-rich green tea extract, acarbose, and epigallocatechin gallate for rat maltase were 0.035 ± 0.005 mg/mL, 0.42 ± 0.02 μM, and 14.0 ± 2.0 μM, respectively. Furthermore, the IC_50_ values of polyphenol-rich green tea extract, acarbose, and epigallocatechin gallate for rat sucrase were 1.8 ± 0.3 mg/mL, 12.3 ± 0.6 μM, and 950 ± 86 μM, respectively. In addition, quercetin showed inhibitory activities with IC_50_ values of 3.5 ± 0.3 mM against rat sucrose [21]. Furthermore, mogroside IV (IC_50_ = 12 mM), siamenoside I (IC_50_ = 10 mM), and mogroside III (IC_50_ = 1.6 mM) isolated from *Siraitia grosvenori* also have maltase inhibitory activity [23]. Kim et al. [24] isolated a bromophenol, bis (2,3-dibromo-4,5-dihydroxybenzyl)ether, from the red alga *Polyopes lancifolia*. That compound had an IC_50_ value of 1.00 ± 0.03 mM against sucrose in rats. According to the above results, B2-3’-*O*-gallate, ECG, and EC have the potential to be maltase and sucrase inhibitors.

### 3.2. Mode of Inhibition of B2-3’-O-gallate, ECG, EC, and Quercetin Toward Maltase

**Figure 3:**
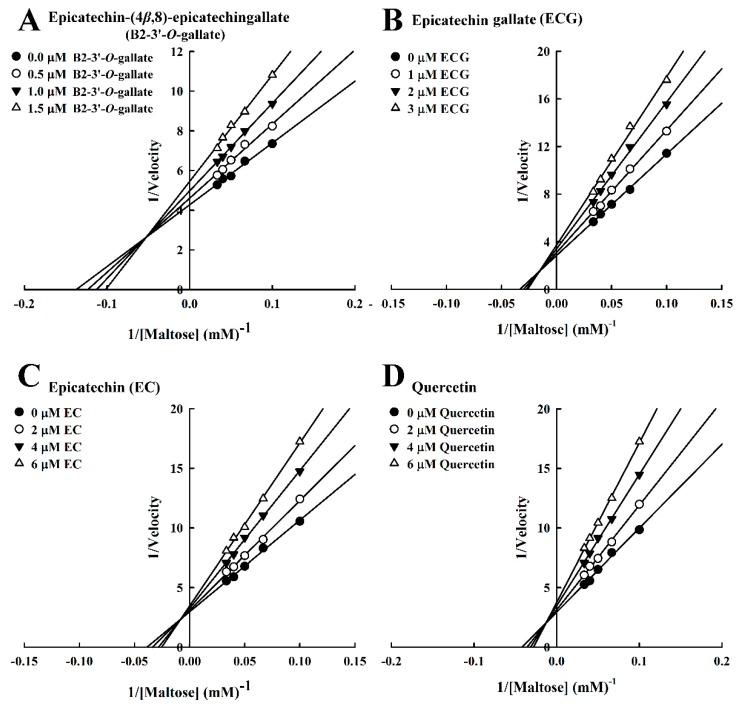
Lineweaver–Burk plots for the inhibition of maltase by maltase inhibitors, with maltose as the substrate. (**A**) B2-3’-*O*-gallate. (**B**) ECG. (**C**) EC. (**D**) Quercetin.

We also evaluated the inhibition kinetics of B2-3’-*O*-gallate, ECG, EC, and quercetin toward maltase. In this study, the Michaelis constant (*K*m) of maltase was 1.01 mM, whereas in the study by Pyner et al. [22], the *K*m value of maltase was 7.5 mM. The Lineweaver–Burk plots of B2-3’-*O*-gallate (Figure 3A), ECG (Figure 3B), EC (Figure 3C), and quercetin (Figure 3D) did not intersect the *x*- or *y*-axis, which is indicative of mixed-type inhibition. A number of mixed-type maltase inhibitors have previously been reported. In a previous study, kinetic analysis revealed that maltase was inhibited by cinnamic acid derivatives, including caffeic acid, ferulic acid, and isoferulic acid, in a mixed-inhibition manner [16]. Our previous results indicated that B2-3’-*O*-gallate (*K*i = 0.30 ± 0.03 μM, *K*i’ = 1.42 ± 0.01 μM), ECG (*K*i = 0.21 ± 0.04 μM, *K*i’ = 2.34 ± 0.06 μM), and quercetin (*K*i = 1.44 ± 0.04 μM, *K*i’ = 9.33 ± 0.10 μM) were mixed-competitive inhibitors of α-glucosidase [15]. Furthermore, a Lineweaver–Burk plot indicated that epigallocatechin gallate was a competitive inhibitor against maltose substrate for maltase, and the *K*i calculated from a Dixon plot was 5.93 ± 0.26 µM [25].

**Figure 4:**
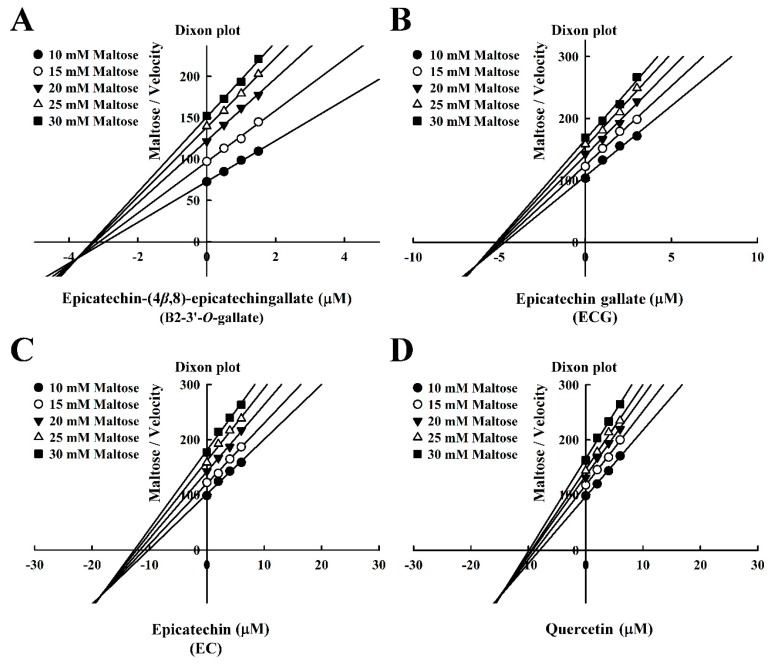
Dixon plots for the inhibition of maltase by maltase inhibitors, with maltose as the substrate. (**A**) B2-3’-*O*-gallate. (**B**) ECG. (**C**) EC. (**D**) Quercetin.

The Dixon plots in Figure 4 revealed that all of the compounds were mixed-type inhibitors of maltase with the following *K*i values: B2-3’-*O*-gallate (1.99 ± 0.02 µM; Figure 4A), ECG (3.14 ± 0.04 µM; Figure 4B), EC (7.02 ± 0.26 µM; Figure 4C), and quercetin (7.81 ± 0.10 µM; Figure 4D). The strength of the *K*i values was as follows: B2-3’-*O*-gallate < ECG < EC < quercetin. The *Ki*’ values were as follows: B2-3’-*O*-gallate (3.73 ± 0.08 µM), ECG (6.38 ± 0.04 µM), EC (18.88 ± 0.22 µM), and quercetin (14.97 ± 0.75 µM). Note that all of the *K*i values were lower than the *K*i’ values. As mentioned previously, *K*i is the equilibrium constant for the inhibition of binding to maltase, whereas *K*i’ is the equilibrium constant for the inhibition of binding to the maltase–maltose complex. In a previous report by Cortés et al. [26], *K*i values are smaller than *K*i’ values in cases of reversible mixed-competitive inhibition. This is an indication that inhibitor–enzyme binding affinity exceeds inhibitor–enzyme–substrate binding affinity, resulting in the mixed-competitive inhibition of maltase. These findings suggest that B2-3’-*O*-gallate, ECG, EC, and quercetin may bind to either maltase or the maltase–maltose complex. Nonetheless, the binding sites and underlying mechanisms of inhibition have yet to be elucidated.

### 3.3. Mode of Inhibition of B2-3’-O-gallate, ECG, EC, and Quercetin Toward Sucrase

**Figure 5:**
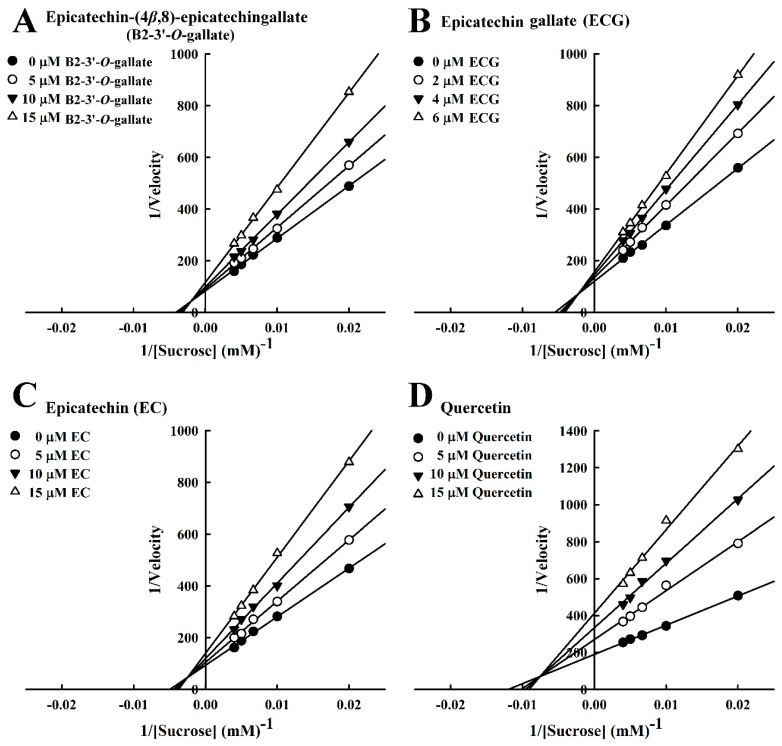
Lineweaver–Burk plots for the inhibition of sucrase by sucrase inhibitors, with sucrose as the substrate. (**A**) B2-3’-*O*-gallate. (**B**) ECG. (**C**) EC. (**D**) Quercetin.

In our analysis of the inhibition kinetics of B2-3’-*O*-gallate, ECG, EC, and quercetin toward sucrose, the Michaelis constant (*K*m) was 100.4 mM. In a previous report by Pyner et al. [22], the *K*m value of sucrase activity was 9.5 mM. The Lineweaver–Burk plots of B2-3’-*O*-gallate (Figure 5A), ECG (Figure 5B), EC (Figure 5C), and quercetin (Figure 5D) did not intersect the *x*- or *y*-axis, which is indicative of mixed-type inhibition. In previous studies, a number of mixed-type sucrase inhibitors have been reported. Cyaniding-3-rutinoside (a natural anthocyanin) was found in litchi and sweet cherry. Kinetics analysis revealed that sucrase was inhibited by cyanidin-3-rutinoside in a mixed-type manner [27]. In addition, ferulic acid and isoferulic acid inhibited sucrase in a mixed-type manner [16]. Furthermore, a Lineweaver–Burk plot indicated that valienamine was a competitive inhibitor against the sucrose substrate of sucrase, and the *K*i calculated from a Dixon plot was 0.77 µM [12].

**Figure 6:**
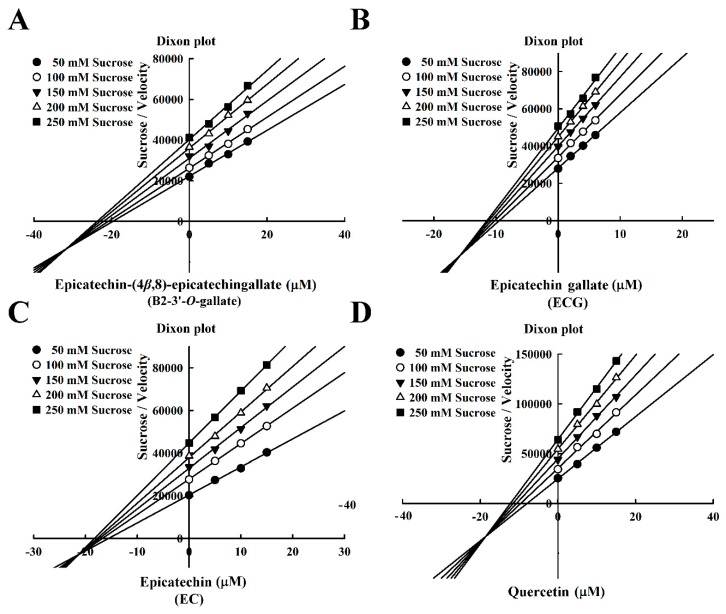
Dixon plots for the inhibition of sucrase by sucrase inhibitors, with sucrose as the substrate. (**A**) B2-3’-*O*-gallate. (**B**) ECG. (**C**) EC. (**D**) Quercetin.

The Dixon plots in Figure 6 indicated that these compounds were mixed-type inhibitors of sucrase with the following *K*i values: B2-3’-*O*-gallate (6.05 ± 0.04 µM; Figure 6A), ECG (8.58± 0.08 µM; Figure 6B), EC (13.72 ± 0.15 µM; Figure 6C), and quercetin (14.05 ± 0.03 µM; Figure 6D). The strength of the *K*i values was as follows: B2-3’-*O*-gallate < ECG < EC < quercetin. The *K*i’ values were as follows: B2-3’-*O*-gallate (31.84 ± 0.34 µM), ECG (15.74 ± 0.32 µM), EC (21.36 ± 0.30 µM), and quercetin (18.54 ± 0.07 µM). Note that the *K*i values of B2-3’-*O*-gallate, ECG, EC, and quercetin were lower than the *K*i’ values, indicating that these compounds were mixed-competitive inhibitors of sucrase. These findings suggest that B2-3’-*O*-gallate, ECG, EC, and quercetin may bind to either sucrase or the sucrase–sucrose complex. Nonetheless, the binding sites and underlying mechanisms of inhibition have yet to be elucidated.

### 3.4. Inhibition Scheme of B2-3’-O-gallate, ECG, EC, and Quercetin for Maltase and Sucrase

**Figure 7:**
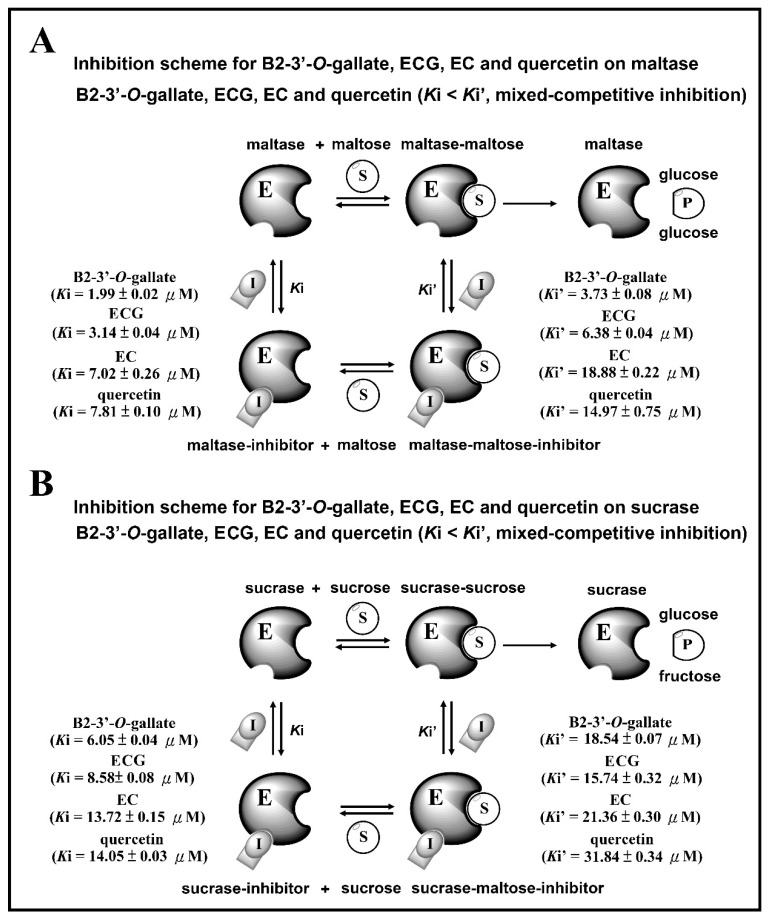
Inhibition schemes for B2-3’-*O*-gallate, ECG, EC, and quercetin toward maltase (**A**) and sucrase (**B**). E: enzyme, S: substrate, I: inhibitor, P: product.

Figure 7 presents the inhibition schemes of the maltase and sucrase inhibitors (B2-3’-*O*-gallate, ECG, EC, and quercetin). Figure 7A shows that maltase hydrolyzes carbohydrates by acting on 1,4-α linkages. Maltase inhibitors counter enzymes in the gut to restrain the liberation of glucose from oligosaccharides and disaccharides, resulting in a reduction in postprandial glucose levels [4]. In the maltase inhibitory activity assay, maltase could hydrolyze maltose to glucose, while maltase inhibitors prevent this maltase activity. *K*i and *K*i’ are the dissociation constants of the maltase–inhibitor complex and the maltase–maltose–inhibitor complex, respectively. According to our results, B2-3’-*O*-gallate (*K*i = 1.99 ± 0.02 µM, *K*i’ = 3.73 ± 0.08 µM), ECG (*K*i = 3.14 ± 0.04 µM, *K*i’ = 6.38 ± 0.04 µM), EC (*K*i = 7.02 ± 0.26 µM, *K*i’ = 18.88 ± 0.22 µM), and quercetin (*K*i = 7.81 ± 0.10 µM, *K*i’ = 14.97 ± 0.75 µM) are mixed-competitive inhibitors of maltase. Note that mixed-competitive inhibitors bind at sites that are distinct from maltose active sites; however, they bind to either maltase or the maltase–maltose complex. Moreover, a similar inhibition scheme was also observed in the results of B2-3’-*O*-gallate, ECG, EC, and quercetin against sucrase (Figure 7B). In the sucrase inhibitory activity assay, sucrase could hydrolyze sucrose to glucose, while sucrase inhibitors prevent this sucrase activity [28]. *K*i and *K*i’ are the dissociation constants of the sucrase–inhibitor complex and the sucrase–sucrose–inhibitor complex, respectively. According to our results, B2-3’-*O*-gallate (*K*i = 6.05 ± 0.04 µM, *K*i’ = 18.54 ± 0.07 µM), ECG (*K*i = 8.58 ± 0.08 µM, *K*i’ = 15.74 ± 0.32 µM), EC (*K*i = 13.72 ± 0.15 µM, *K*i’ = 21.36 ± 0.30 µM), and quercetin (*K*i = 14.05 ± 0.03 µM, *K*i’ = 31.84 ± 0.34 µM) are mixed-competitive inhibitors of sucrase. Mixed-competitive inhibitors bind at sites that are distinct from sucrose active sites; however, they bind to either sucrase or the sucrase–sucrose complex. Note that B2-3’-*O*-gallate, ECG, and EC all had significant inhibitory effects on maltase as well as sucrase.

## 4. Conclusions

B2-3’-*O*-gallate, ECG, and EC isolated from *R. crenulata* displayed maltase and sucrase inhibitory properties in a mixed-competitive mode. The IC_50_ values of these compounds for both maltase and sucrose were in the following order: EC > ECG > B2-3’-*O*-gallate. Among these compounds, B2-3’-*O*-gallate had the highest maltase and sucrose inhibitory activities. We observed that the IC_50_ values of B2-3’-*O*-gallate, ECG, and EC were lower than those of quercetin, which means that they have greater potential for the prevention of hyperglycemia associated with maltase or sucrase. Our findings clearly identify B2-3’-*O*-gallate, ECG, and EC as likely candidates for the treatment of diabetes mellitus, warranting further in vivo studies.

## Figures and Tables

**Table 1:** Structure, molecular weight, molecular formula, and IC_50_ values of maltase and sucrase inhibitors.

Inhibitor	Structure	Molecular Weight	Molecular Formula	IC_50_ (μM) ^a^	
				Maltase	Sucrase
Epicatechin-(4*β*,8)-epicatechingallate (B2-3’*-O-*gallate)	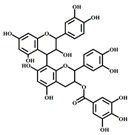	731.16	C_37_H_30_O_16_	1.73 ± 1.37	6.91 ± 3.41
Epicatechin gallate (ECG)	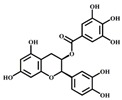	442.37	C_22_H_18_O_10_	3.64 ± 2.99	18.27 ± 3.99
Epicatechin (EC)	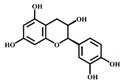	290.27	C_15_H_14_O_6_	6.25 ± 1.84	18.91 ± 3.66
Quercetin	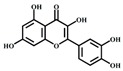	302.24	C_15_H_10_O_7_	8.33 ± 3.91	18.98 ± 2.53

^a^ IC_50_ values are expressed as the mean ± standard deviation.
